# Antibiotic-Resistant *Neisseria gonorrhoeae* Spread Faster with More Treatment, Not More Sexual Partners

**DOI:** 10.1371/journal.ppat.1005611

**Published:** 2016-05-19

**Authors:** Stephanie M. Fingerhuth, Sebastian Bonhoeffer, Nicola Low, Christian L. Althaus

**Affiliations:** 1 Institute of Integrative Biology, ETH Zurich, Zurich, Switzerland; 2 Institute of Social and Preventive Medicine (ISPM), University of Bern, Bern, Switzerland; Emory University, UNITED STATES

## Abstract

The sexually transmitted bacterium *Neisseria gonorrhoeae* has developed resistance to all antibiotic classes that have been used for treatment and strains resistant to multiple antibiotic classes have evolved. In many countries, there is only one antibiotic remaining for empirical *N. gonorrhoeae* treatment, and antibiotic management to counteract resistance spread is urgently needed. Understanding dynamics and drivers of resistance spread can provide an improved rationale for antibiotic management. In our study, we first used antibiotic resistance surveillance data to estimate the rates at which antibiotic-resistant *N. gonorrhoeae* spread in two host populations, heterosexual men (HetM) and men who have sex with men (MSM). We found higher rates of spread for MSM (0.86 to 2.38 y^−1^, mean doubling time: 6 months) compared to HetM (0.24 to 0.86 y^−1^, mean doubling time: 16 months). We then developed a dynamic transmission model to reproduce the observed dynamics of *N. gonorrhoeae* transmission in populations of heterosexual men and women (HMW) and MSM. We parameterized the model using sexual behavior data and calibrated it to *N. gonorrhoeae* prevalence and incidence data. In the model, antibiotic-resistant *N. gonorrhoeae* spread with a median rate of 0.88 y^−1^ in HMW and 3.12 y^−1^ in MSM. These rates correspond to median doubling times of 9 (HMW) and 3 (MSM) months. Assuming no fitness costs, the model shows the difference in the host population’s treatment rate rather than the difference in the number of sexual partners explains the differential spread of resistance. As higher treatment rates result in faster spread of antibiotic resistance, treatment recommendations for *N. gonorrhoeae* should carefully balance prevention of infection and avoidance of resistance spread.

## Author Summary

More and more infectious disease treatments fail because the causative pathogens are resistant to the drugs used for treatment. For the treatment of *Neisseria gonorrhoeae*, a sexually transmitted bacterium, drug resistance is a particularly big problem: there is only a single antibiotic left that is recommended for treatment. We aimed to understand how antibiotic-resistant *N. gonorrhoeae* spread in a sexually active host population and how the spread of resistance can be slowed. From antibiotic resistance surveillance data, we first estimated the rate at which antibiotic-resistant *N. gonorrhoeae* spread. Second, we reproduced the observed dynamics in a mathematical model describing the transmission between hosts. We found that antibiotic-resistant *N. gonorrhoeae* spread faster in host populations of men who have sex with men than in host populations of heterosexuals. We could attribute the faster spread of resistant pathogens to higher treatment rates. This finding implies that promoting screening to control antibiotic-resistant *N. gonorrhoeae* could in fact accelerate their spread.

## Introduction

Antibiotic-resistant *Neisseria gonorrhoeae* can evolve and spread rapidly [1]. Resistance is commonly observed against the antibiotic classes penicillin, tetracycline and fluoroquinolones [2–4]. Resistance also emerged against cefixime, an oral third generation cephalosporin, in recent years [2, 3]. Since 2010, cefixime is no longer recommended as first-line treatment [5] following guidelines from the World Health Organization (WHO) that an antibiotic should not be used when more than 5% of *N. gonorrhoeae* isolates are resistant [6]. Injectable ceftriaxone, in combination with oral azithromycin, is now the last antibiotic remaining as recommended first-line treatment [7]. Although other antibiotics are being tested for their safety and efficacy for *N. gonorrhoeae* treatment [8], no new classes of antibiotics are currently available [4] and management of antibiotics is urgently needed to preserve their efficacy. The current management strategy tries to reduce the overall burden of *N. gonorrhoeae* infection by expanded screening and treatment of hosts [9, 10], but the outcome of this strategy for resistance is uncertain. Understanding the drivers of resistance spread and anticipating future resistance trends will provide rationales for antibiotic management and help to improve antibiotic treatment strategies.

Men who have sex with men (MSM) are host populations that have higher levels of antibiotic-resistant *N. gonorrhoeae* than heterosexual host populations [3]. In a study [5] based on the Gonococcal Resistance to Antimicrobials Surveillance Programme (GRASP) in England and Wales, cefixime-resistant *N. gonorrhoeae* were mainly found in MSM until 2011. The authors suggested that cefixime resistance was circulating in a distinct sexual network of highly active MSM and that bridging between MSM and heterosexuals was necessary for subsequent spread among heterosexual hosts. However, cefixime-resistant *N. gonorrhoeae* might have already been spreading undetected in the heterosexual host population.

Mathematical models can help explain the differential observations of antibiotic-resistant *N. gonorrhoeae* in different host populations. In 1978, Yorke et al. [11] introduced the concept of core groups to model the transmission of *N. gonorrhoeae*. The concept of core groups posits that an infection can only be maintained in a host population if a highly sexually active group of hosts is responsible for a disproportionate amount of transmissions. More recent modeling studies have examined the transmission of antibiotic-resistant *N. gonorrhoeae*. Chan et al. [12] found that prevalence rebounds more quickly to a pre-treatment baseline when treatment is focused on the core group. Xiridou et al. [13] developed a *N. gonorrhoeae* transmission model to determine the impact of different treatment strategies on the prevalence of *N. gonorrhoeae* in Dutch MSM. They found that increased treatment rates could increase the spread of resistance, whereas re-treatment could slow it down. Hui et al. [14] used an individual-based *N. gonorrhoeae* transmission model in a heterosexual host population to investigate the effect of a molecular resistance test on the time until 5% resistance are reported. None of these studies has investigated or explained the differences in the spread of antibiotic-resistant *N. gonorrhoeae* in MSM and heterosexual host populations.

In this study, we investigated the dynamics and determinants of antibiotic-resistant *N. gonorrhoeae* spread using surveillance data and mathematical modeling. We estimated the rates at which resistance spreads in heterosexual men (HetM) and MSM using surveillance data from the USA and from England and Wales. We then developed a mathematical model of *N. gonorrhoeae* transmission to reconstruct the observed dynamics of resistance spread. This allowed us to determine the major driver of resistance spread, and to explore the expected rates at which resistance spreads in MSM and heterosexual host populations.

## Methods

### Data

#### Data sources

We used data from the GRASP [15, 16] and the Gonococcal Isolate Surveillance Project (GISP) [17]. GRASP is a program of Public Health England (PHE) that monitors antibiotic-resistant *N. gonorrhoeae* in England and Wales. GISP is an equivalent program from the Centers for Disease Control and Prevention (CDC) in the USA. We used Plot Digitizer 2.6.6 [18] to digitize data on the proportion of cefixime- and ciprofloxacin-resistant *N. gonorrhoeae* from figures that were published online (see S1 and S2 Tables).

#### Rate of spread

We determined the rate of resistance spread by assuming that the proportion of antibiotic-resistant *N. gonorrhoeae* follows logistic growth. We used the least squares function *nls* from the R software environment for statistical computing [19] to fit the following function to the data:

$$f\left(t\right)=\frac{c}{1+a\times exp(-bt)}.$$

*f*(*t*) represents the proportion of antibiotic-resistant *N. gonorrhoeae* at time *t*, *c* is the maximal proportion of antibiotic-resistant *N. gonorrhoeae* (carrying capacity), *a* is the ratio between antibiotic-sensitive and -resistant *N. gonorrhoeae* at time 0, and *b* is the rate at which the proportion of antibiotic-resistant *N. gonorrhoeae* increases in the initial exponential growth phase. We only used data from the years before the first decline in the proportion of resistant *N. gonorrhoeae* because we were interested in the rate of resistance spread during the initial exponential growth phase and while the antibiotic was still used.

### Model

#### Transmission model

We developed a mathematical model to describe the spread of antibiotic-resistant *N. gonorrhoeae* in a given host population [12]:

$$\begin{array}{lll} {\dot{S}}_{i} & = & -S_{i}\pi_{i}\sum_{j\in G}\rho_{ij}\beta_{ij}\frac{I_{Sen_{j}}+I_{Res_{j}}}{N_{j}}+\nu (I_{Sen_{i}}+I_{Res_{i}})+\tau (1-\mu )I_{Sen_{i}}\\ & - & \alpha S_{i}+\alpha N_{i}-\gamma S_{i}+\gamma N_{i}\sum_{j\in G}S_{j},\\ {\dot{I}}_{Sen_{i}} & = & S_{i}\pi_{i}\sum_{j\in G}\rho_{ij}\beta_{ij}\frac{I_{Sen_{j}}}{N_{j}}-\nu I_{Sen_{i}}-\tau I_{Sen_{i}}\\ & - & \alpha I_{Sen_{i}}-\gamma I_{Sen_{i}}+\gamma N_{i}\sum_{j\in G}I_{Sen_{j}},\\ {\dot{I}}_{Res_{i}} & = & S_{i}\pi_{i}\sum_{j\in G}\rho_{ij}\beta_{ij}\frac{I_{Res_{j}}}{N_{j}}-\nu I_{Res_{i}}+\tau \mu I_{Sen_{i}}\\ & - & \alpha I_{Res_{i}}-\gamma I_{Res_{i}}+\gamma N_{i}\sum_{j\in G}I_{Res_{j}}. \end{array}$$

**Fig 1:**
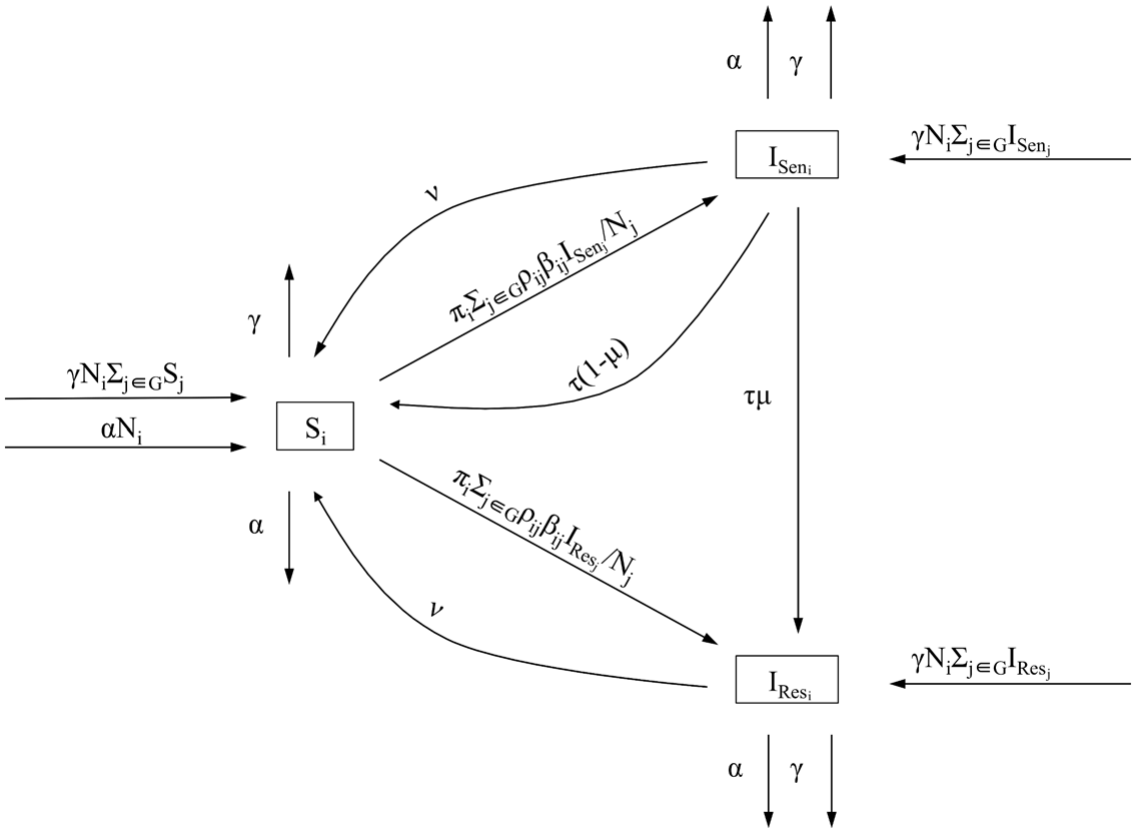
Structure of *N. gonorrhoeae* transmission model. *N*_*i*_ sexual activity group *i*, *S*_*i*_ susceptible hosts, *I*_*Sen*_*i*__ hosts infected with antibiotic-sensitive strain, *I*_*Res*_*i*__ hosts infected with antibiotic-resistant strain, *π*_*i*_ partner change rate, *β*_*ij*_ transmission probability per partnership, *ρ*_*ij*_ mixing between and within sexual activity groups, *τ* treatment rate, *ν* spontaneous recovery rate, *μ* probability of resistance during treatment, *α* rate of entering and leaving the population, *γ* redistribution rate, *G* set of low and high sexual activity groups.

*Sen* and *Res* indicate the antibiotic-sensitive and -resistant *N. gonorrhoeae* strains, *G* = {*L*, *H*} is the set of low and high sexual activity groups and *i* ∈ *G* (Fig 1). Each sexual activity group *N*_*i*_ consists of susceptible hosts, *S*_*i*_, hosts infected with an antibiotic-sensitive strain, *I*_*Sen*_*i*__, and hosts infected with an antibiotic-resistant strain, *I*_*Res*_*i*__. To account for individual heterogeneity in sexual behavior [20], hosts are redistributed to either the same or the other sexual activity group at rate *γ*. Redistribution is proportional to the size of the sexual activity group, i.e. hosts from the larger sexual activity group are less likely to change their sexual behavior than hosts from the smaller sexual activity group. Hosts can also leave or enter the population at rate *α*. Susceptible hosts become infected depending on the partner change rate, *π*_*i*_, the transmission probability per partnership, *β*_*ij*_, and the sexual mixing matrix, *ρ*_*ij*_, which describes how many partnerships are formed within and outside the host’s activity group:

$$\rho_{ij}=\epsilon \delta_{ij}+\left(1-\epsilon \right)\frac{\pi_{j}N_{j}}{\sum_{k\in G}\pi_{k}N_{k}},$$

where *δ*_*ij*_ = 1 if *i* = *j* and *δ*_*ij*_ = 0 if *i* ≠ *j*. *ϵ* is the sexual mixing coefficient [21]. It ranges from 0 (random or proportionate mixing) to 1 (assortative mixing, i.e. all partnerships are formed with hosts from same group). Hosts infected with an antibiotic-sensitive strain can recover spontaneously at rate *ν* or receive treatment at rate *τ*. Treatment occurs both when the host seeks treatment for a symptomatic infection or is screened and diagnosed with an asymptomatic infection. Hosts receiving treatment recover at rate *τ*(1 − *μ*) and develop resistance during treatment with probability *μ*. Hosts infected with an antibiotic-resistant strain can only recover spontaneously at rate *ν*. We assumed equal fitness of antibiotic-sensitive and -resistant strains in absence of treatment, i.e. no fitness costs for the antibiotic-resistant strain. We evaluated the impact of fitness costs on the model outcomes in a sensitivity analysis (see S1 Appendix).

#### Parameters

Model parameters were estimated from sexual behavior data, calibrated through model simulation or informed by literature. The partner change rate and the proportions of the host population in each sexual activity group were estimated from the second British National Survey of Sexual Attitudes and Lifestyles (Natsal-2) [22], a population-based cross-sectional survey. For the heterosexual men and women (HMW) model population, we used the number of new heterosexual partners in the last year of all male and female participants between 16–44 years who reported never having had a homosexual partner. For the MSM model population, we used the number of new homosexual partners in the last year of all male participants between 16–44 years who reported ever having had a homosexual partner. For each host population, the number of partners per year was weighted with weights provided in Natsal-2 to adjust for unequal selection probabilities in the survey. We estimated the partner change rate by assuming that the reported numbers of new sexual partners can be described by two Poisson distributions with means *π*_*L*_ and *π*_*H*_, weighted by the proportion of individuals in each sexual activity group [23]. For HMW, the sexual partner change rates are *π*_*L*_ = 0.25 y^−1^ and *π*_*H*_ = 4.57 y^−1^ with *N*_*H*_ = 6.3% of the population being in the high sexual activity group and *N*_*L*_ = 1 − *N*_*H*_. The obtained partner change rates for MSM are *π*_*L*_ = 0.41 y^−1^ and *π*_*H*_ = 30.49 y^−1^ with *N*_*H*_ = 5.3% of the population belonging to the high sexual activity group and *N*_*L*_ = 1 − *N*_*H*_.

We calibrated the sexual mixing coefficient, *ϵ*, the fraction of diagnosed and treated infections, *ϕ*, the average duration of infection, *D*, and the per partnership transmission probabilities within the low, *β*_*LL*_, and the high sexual activity group, *β*_*HH*_, to *N. gonorrhoeae* prevalence and incidence using the following algorithm:

1. Define prior parameter distributions (Table 1).
2. Define the ranges for the expected prevalence and incidence of diagnosed infections (Table 2) of urethral and cervical *N. gonorrhoeae* infections for HMW, and urethral, rectal and pharyngeal infections for MSM.
3. Randomly draw 10^7^ parameters sets from prior distributions.
4. Simulate the transmission model until it approaches a resistance-free (*μ* = 0) endemic equilibrium using the ordinary differential equation solver *runsteady* from the R [19] package *rootSolve* [24].
5. Select the parameters sets (posterior distributions) that result in prevalences and incidences within the defined range.

**Table 1:** Prior distributions and posterior estimates of model parameters.

parameter	description	priors	M_*MSM*_	IQR_*MSM*_	M_*HMW*_	IQR_*HMW*_
*ϵ*	sexual mixing coefficient	U(0,1)	0.57	0.30–0.80	0.73	0.53–0.89
*ϕ*	fraction of diagnosed and treated infections	U(0,1)	0.64	0.48–0.81	0.50	0.36–0.66
*D*	average duration of infection (years)	Γ(2, 0.125)	0.19	0.14–0.25	0.55	0.46–0.66
*β*_*LL*_	transmission probability within low activity group	U(0,1)	0.59	0.42–0.77	0.87	0.79–0.94
*β*_*HH*_	transmission probability within high activity group	$U(0,\beta_{LL})$	0.30	0.25–0.40	0.72	0.63–0.81

We assumed that the duration of infection is described by a gamma distribution Γ(*k*, *θ*) with shape parameter *k* = 2 and scale parameter *θ* = 0.125 y resulting in an average infectious duration of 0.25 y. Because highly sexually active hosts have fewer sex acts per partnership, we assumed that the transmission probability within the high activity group cannot be higher than the transmission probability within the low activity group. M and IQR represent the median and interquartile range of the posterior distributions.

**Table 2:** Prevalence and incidence ranges used for model calibration.

parameter	infection site	host population	sexual activity group	range
prevalence	urethral, cervical	HMW	low	0–0.38%
prevalence	urethral, cervical	HMW	high	0.16–100%
prevalence	urethral, cervical	HMW	either	0.16–0.38%
prevalence	pharyngeal, anal, urethral	MSM	low	0–2.79%
prevalence	pharyngeal, anal, urethral	MSM	high	1.19–100%
prevalence	pharyngeal, anal, urethral	MSM	either	1.19–2.79%
incidence	urethral, cervical	HMW	either	0.12–0.36% person^−1^ y^−1^
incidence	pharyngeal, anal, urethral	MSM	either	5.88–7.19% person^−1^ y^−1^

Prevalence and incidence ranges for HMW were based on the National Health and Nutrition Examination Survey [26] and surveillance data [27], both from CDC. For MSM, prevalence and incidence ranges were based on the Health in Men Study in Australia [28, 29]. The upper and lower bound of the ranges for the low and high sexual activity groups are given by the lower and upper bound of the overall population.

Information about parameter estimates for *N. gonorrhoeae* is scarce, so we chose to use non-informative priors for all parameters except the duration of infection which was informed by Garnett et al. [25]. The ranges for the expected prevalence and incidence of diagnosed infections in HMW were based on the National Health and Nutrition Examination Survey [26] and surveillance data [27], both from CDC. For MSM, we used data from the Health in Men Study in Australia [28, 29]. We compared the model predicted prevalence and incidence of diagnosed infections to the prevalence and incidence from data without allowing for resistance in the simulations, because we assumed the data were collected when treatment was mostly effective. We calculated the model incidence of diagnosed and treated infections for sexual activity group *i* with $\phi S_{i}\pi_{i}\sum_{j\in G}\rho_{ij}\beta_{ij}\frac{I_{Sen_{j}}+I_{Res_{j}}}{N_{j}}$ per year.

We set the rate of entering and leaving the population, $\alpha =\frac{1}{29}\;{\text{y}}^{-1}$, because we only considered hosts 16–44 years of age. Since the sexual partner change rates are based on the numbers of new sexual partners within the last year, we assumed that hosts stay on average one year (*γ* = 1 y^−1^) in their sexual activity group before they are redistributed to either the same or the other sexual activity group [30]. We do not have information on the probability of resistance during treatment. We set the probability of resistance during treatment to *μ* = 10^−3^ and performed a sensitivity analysis to assess the impact of *μ* on the model outcomes.

The remaining model parameters (*τ*, *ν*, *β*_*LH*_, *β*_*HL*_) are composites of other parameters (Table 3). Since $D=\frac{1}{\nu +\tau }$ and $\phi =\frac{\tau }{\tau +\nu }$, the treatment rate is $\tau =\frac{\phi }{D}$, and the spontaneous recovery rate is $\nu =\frac{1-\phi }{D}$. *β*_*LH*_ and *β*_*HL*_ are the transmission probabilities per partnership between hosts of the high and low activity groups. We assumed that the between-group transmission probabilities are given by the geometric mean of the within-group transmission probabilities.

**Table 3:** Composite model parameters.

parameter	description	formula
*τ*	treatment rate per year	*ϕ*/*D*
*ν*	spontaneous recovery rate per year	$\frac{1-\phi }{D}$
*β*_*LH*_	transmission probability per partnership between low and high sexual activity host	$\sqrt{\beta_{LL}\beta_{HH}}$
*β*_*HL*_	transmission probability per partnership between high an low sexual activity host	$\sqrt{\beta_{LL}\beta_{HH}}$

The composite model parameters *τ* and *ν* relate to other model parameters with $D=\frac{1}{\nu +\tau }$ and $\phi =\frac{\tau }{\nu +\tau }$. We assumed that the transmission probabilities between hosts of different sexual activity groups are given by the geometric mean of the transmission probabilities for hosts within each group.

## Results

**Fig 2:**
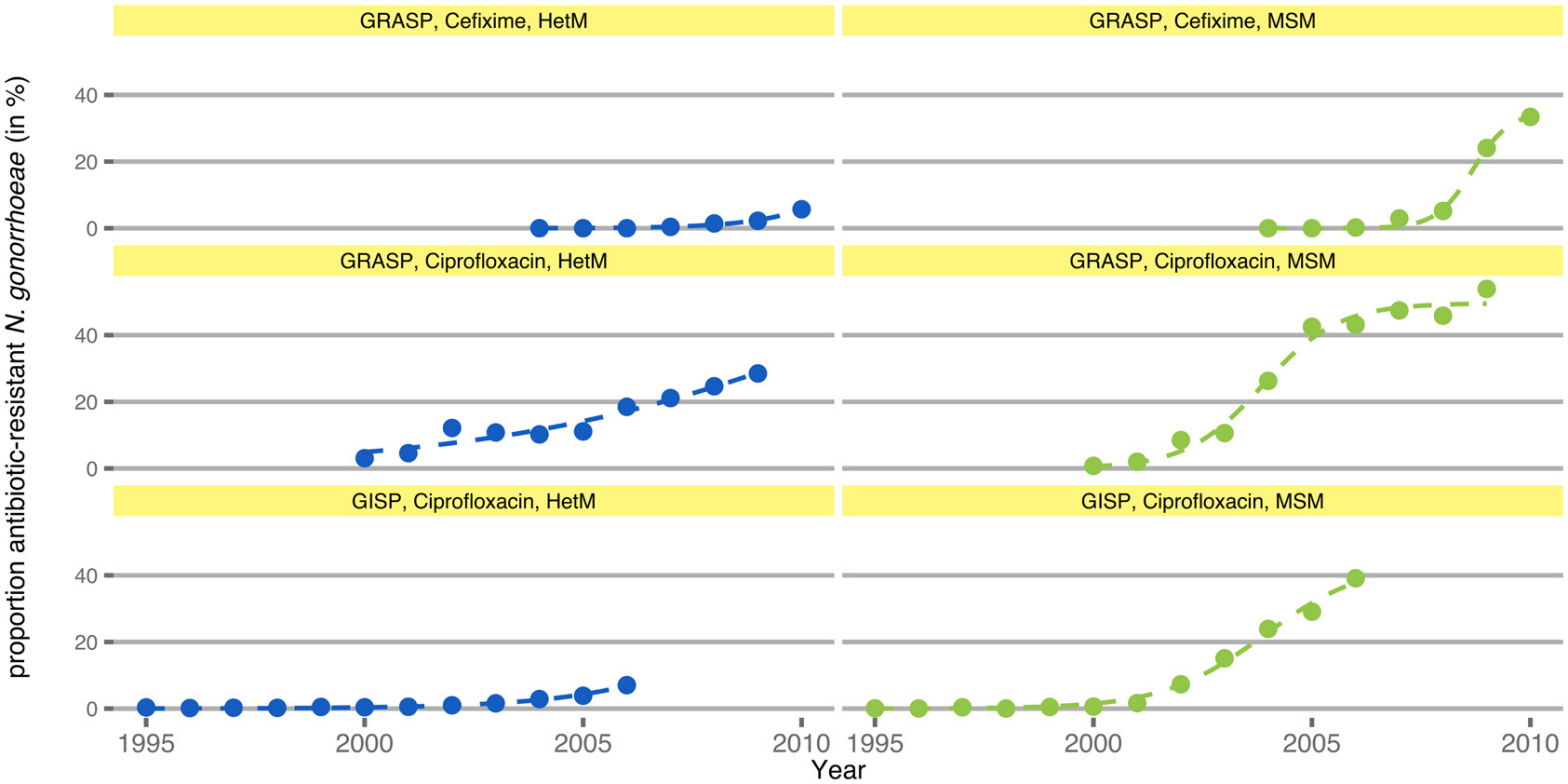
Increase in antibiotic-resistant *N.gonorrhoeae*. Points show data from antibiotic resistance surveillance programs (GRASP and GISP). Dashed lines indicate the fit of the logistic growth model to the data. For a given antibiotic and surveillance program, the rates of spread in MSM (green) are consistently higher than those in HetM (blue).

We fitted a logistic growth model to the proportion of antibiotic-resistant *N. gonorrhoeae* as observed in the two gonococcal surveillance programs (Fig 2). The proportion of cefixime-resistant *N. gonorrhoeae* in GRASP appears to increase for both HetM and MSM after 2006. Ciprofloxacin-resistant *N. gonorrhoeae* in HetM and MSM were spreading in all observed host populations after the year 2000. For a given antibiotic and surveillance program, the rates of resistance spread were consistently higher for MSM than for HetM (Table 4). The average rate of resistance spread was 0.53 y^−1^ for HetM and 1.46 y^−1^ for MSM, corresponding to doubling times of 1.3 y (HetM) and 0.5 y (MSM) during the initial exponential growth phase.

**Table 4:** Rates of resistance spread in *N. gonorrhoeae* surveillance programs.

program	antibiotic	years	host population	rate (95% CI)
GRASP	Cefixime	2004–2010	HetM	0.86 (0.73–1.00) y^−1^
GRASP	Cefixime	2004–2010	MSM	2.38 (1.72–3.03) y^−1^
GRASP	Ciprofloxacin	2000–2009	HetM	0.24 (0.03–0.45) y^−1^
GRASP	Ciprofloxacin	2000–2009	MSM	1.15 (0.76–1.54) y^−1^
GISP	Ciprofloxacin	1995–2006	HetM	0.50 (0.45–0.55) y^−1^
GISP	Ciprofloxacin	1995–2006	MSM	0.86 (0.66–1.06) y^−1^

Estimated rates of resistance spread from the Gonococcal Resistance to Antimicrobials Surveillance Programme (GRASP, England and Wales) and from the Gonococcal Isolate Surveillance Project (GISP, USA). CI: confidence interval.

**Fig 3:**
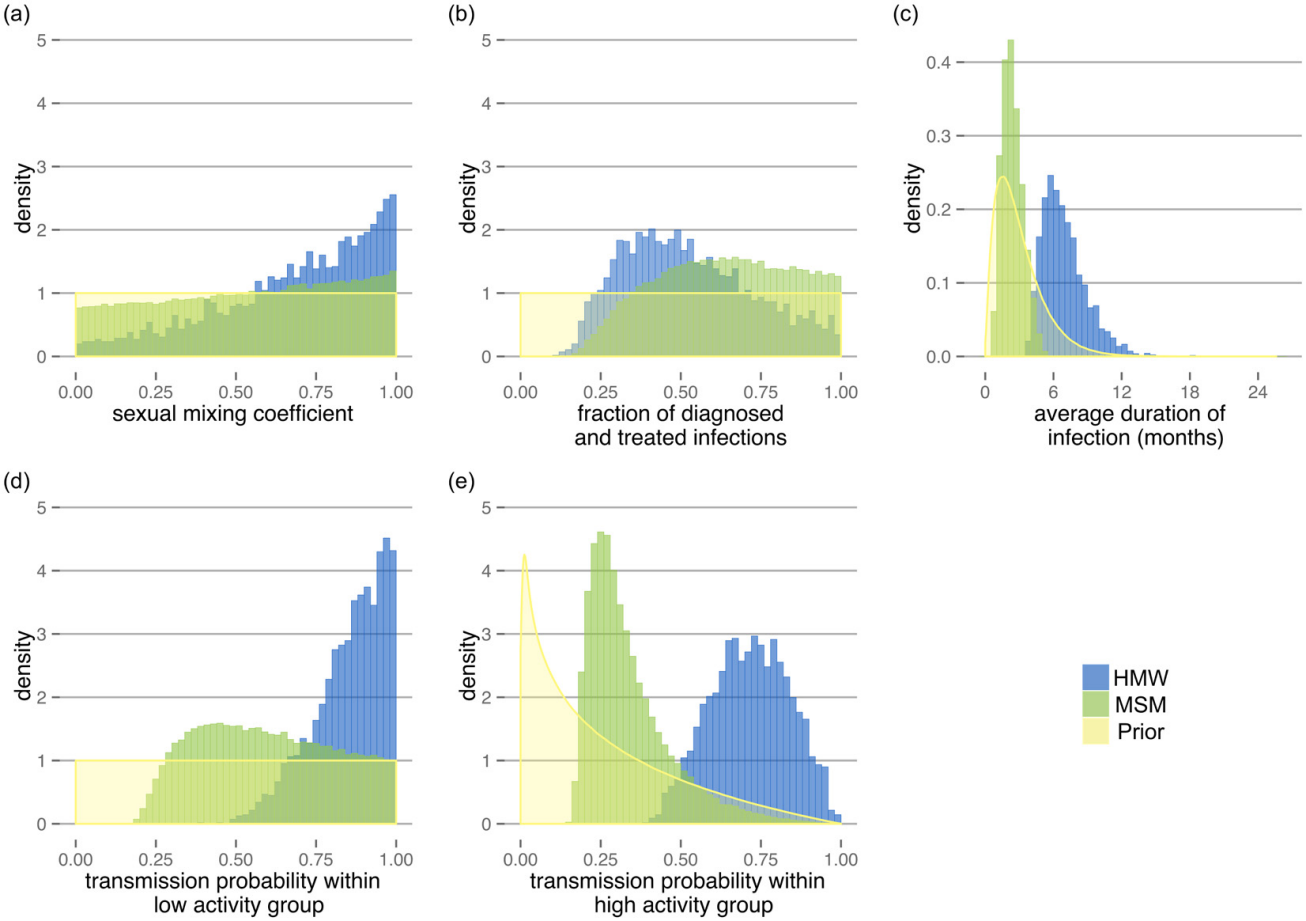
Prior and posterior distributions of the parameters. Prior distributions (yellow) are shown together with posterior distributions for HMW (blue) and MSM (green) for (a) the sexual mixing coefficient, *ϵ*, (b) the fraction of diagnosed and treated infections, *ϕ*, (c) the average duration of infection, *D*, (d) the transmission probability within the low activity group, *β*_*LL*_, and (e) the transmission probability within the high activity group, *β*_*HH*_.

Next, we studied the transmission of *N. gonorrhoeae* and the spread of resistance in the dynamic transmission model. We calibrated five model parameters to expected prevalence and incidence in MSM and HMW host populations. The posterior distributions of the parameters were based on 2,779 parameter sets for HMW and 65,699 parameter sets for MSM (Fig 3, Table 1). Distributions of the modeled prevalence and incidence of diagnosed infections after calibration are provided as Supporting Information (S1 and S2 Figs, S3 Table). The sexual mixing coefficient showed a tendency towards assortative mixing in both MSM and HMW (Fig 3a). The fraction of diagnosed and treated infections tended to be higher in MSM compared to HMW (Fig 3b), whereas the infectious duration was considerably shorter in MSM (median: 2.3 months, IQR: 1.7–3.0 months) than in HMW (median: 6.6 months, IQR: 5.5–7.9 months) (Fig 3c). The transmission probabilities per partnership were generally higher in HMW than in MSM (Fig 3d and 3e).

**Fig 4:**
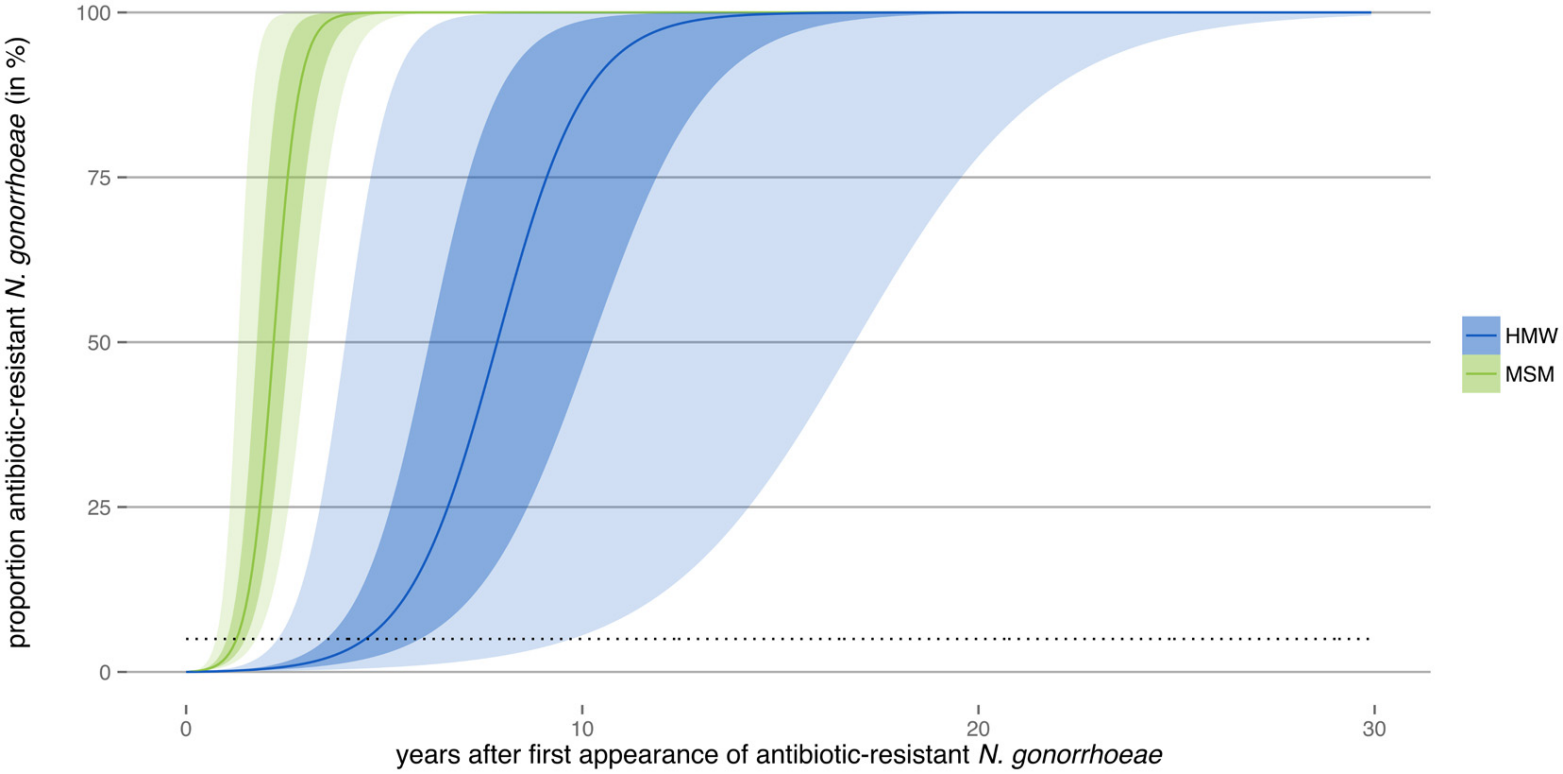
Spread of antibiotic resistance in the transmission model. Ranges indicating 50% of all simulations are shown in dark color, and ranges indicating 95% of all simulations are shown in light color. The continuous lines describe the median proportion of antibiotic-resistant *N. gonorrhoeae* for all simulations. The black dotted line indicates the 5% threshold.

After calibration, we used the dynamic transmission model to study the spread of antibiotic-resistant *N. gonorrhoeae*. The proportion of antibiotic-resistant *N. gonorrhoeae* increased faster in MSM than in HMW (Fig 4). In HMW, the median of all simulations reached 5% resistance in fewer than 4.5 y and 50% resistance in fewer than 7.8 y after appearance of the first antibiotic-resistant *N. gonorrhoeae* infection. In the MSM population, the median of all simulations reached a resistance level of 5% in fewer than 1.7 y and 50% in fewer than 2.6 y after resistance first appears in the population. The range spanned by all simulations was much wider in HMW than in MSM: 95% of all simulations reached the 5% threshold in fewer than 2.7–7.7 y (HMW), compared with 1.1–2.2 y (MSM).

**Fig 5:**
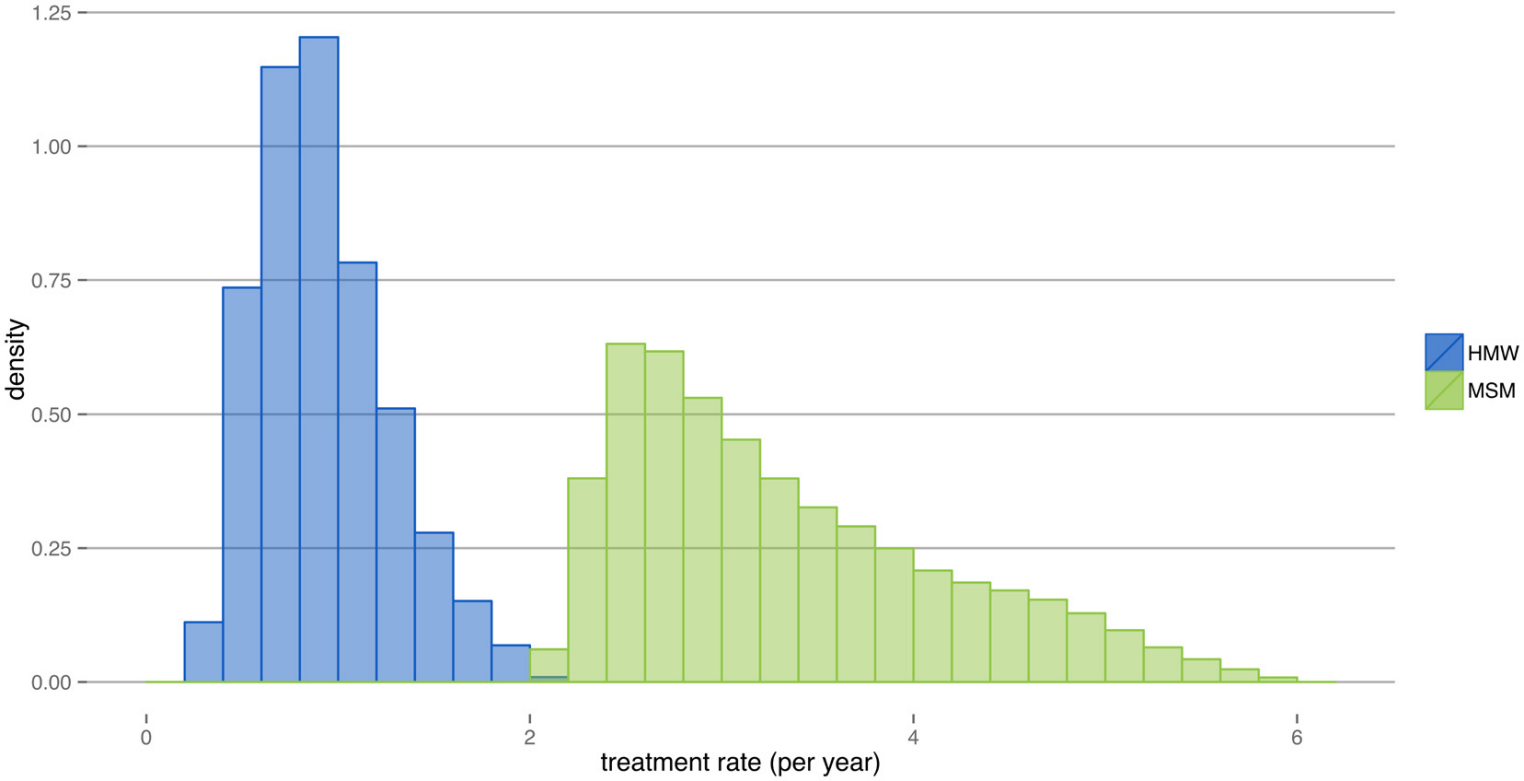
Distribution of treatment rates in HMW and MSM. Treatment rates closely approximate the rates of resistance spread. The median treatment rate was 0.88 y^−1^ in HMW and 3.12 y^−1^ in MSM.

Antibiotic-sensitive and -resistant *N. gonorrhoeae* share the same resource for growth, i.e. the susceptible hosts. The rate at which one strain replaces the other strain in the host population is given by the difference in their net growth rates. We assume that the transmission probabilities and the infectious duration of the two strains are the same. Since the probability of resistance during treatment is very small (*μ* ≪ 1), the difference in net growth rates between the strains is approximated by the treatment rate *τ* and corresponds to the rate of spread of antibiotic-resistant *N. gonorrhoeae*. The observed distributions of treatment rates from the transmission model hardly overlap between HMW and MSM (Fig 5). The median treatment rates, i.e. the approximated median rates of resistance spread in the transmission model are 3.12 y^−1^ (MSM) and 0.88 y^−1^ (HMW).

We tested whether changes in the probability of resistance during treatment, *μ*, and fitness costs in the antibiotic-resistant strain alter the model outcomes. Higher probabilities of resistance during treatment accelerate the establishment of antibiotic-resistant *N. gonorrhoeae* in the population and hence reduce the time until 5% resistance is reached (S3 Fig). Higher probabilities of resistance during treatment, however, do not affect rates of spread, unless the probability of resistance during treatment is unrealistically high (10%) (S4 Fig). Fitness costs in the antibiotic-resistant strain result in rates of resistance spread that are lower than the treatment rate *τ* (Fig. B in S1 Appendix). Fitness costs that reduce the transmission probability per partnership, *β*_*ij*_, have a stronger effect than fitness costs that reduce the duration of infection. The effects of fitness costs are independent of the sexual partner change rate, *π*_*i*_, and *β*_*ij*_ if they affect the duration of infection, but can vary with *π*_*i*_ and *β*_*ij*_ if they affect the transmission probability per partnership (Fig. C in S1 Appendix). While high fitness costs can prevent the spread of antibiotic-resistant strains (Fig. A in S1 Appendix), fitness costs between 0%–10% have only small effects on the rates of resistance spread (Fig. B in S1 Appendix).

## Discussion

In this study, we quantified the rate at which antibiotic-resistant *N. gonorrhoeae* spread in heterosexual and MSM populations. We used data from two different surveillance programs and estimated that the proportion of ciprofloxacin- and cefixime-resistant *N. gonorrhoeae* doubles on average every 1.3 y in HetM and 0.5 y in MSM. The faster spread of antibiotic-resistant *N. gonorrhoeae* in MSM than in heterosexual hosts was corroborated using a dynamic transmission model, which was calibrated to observed prevalence and incidence rates. The model allowed us to identify the higher treatment rates in MSM, compared with heterosexual hosts, as the major driver for the faster spread of antibiotic-resistant *N. gonorrhoeae*.

To our knowledge, this is the first study to have analyzed and interpreted *N. gonorrhoeae* antibiotic resistance surveillance data in a dynamic and quantitative manner. The transmission model was parameterized using sexual behavior data for HMW and MSM from Natsal-2 [22], a large probability sample survey of sexual behavior. Calibrating the model to observed prevalence and incidence rates allowed us to use largely uninformative priors for the model parameters. The calibration makes our model more robust to changes in parameters than using fixed parameter values, especially since for *N. gonorrhoeae* available parameter values are very uncertain [31]. It also allowed us to rely on few assumptions about the natural history of *N. gonorrhoeae* infection.

The limitations to our study need to be taken into consideration when interpreting the findings. First, we used data from different sources, although all were collected in high income countries. The antibiotic resistance surveillance data are from programs in England and Wales and the USA. The mathematical transmission model was parameterized using British sexual behavior data [22] and calibrated to prevalence and incidence rates from the USA (HMW) [26, 27] and Australia (MSM) [28, 29]. For simplicity, we modeled the heterosexual and MSM host populations separately although there is some mixing between them. We assumed the sexual behavior of heterosexual men and women to be the same and pooled their behavioral data. Second, we assumed complete resistance against the antibiotic, i.e. 100% treatment failure. We further assumed that treatment of the sensitive strain is 100% efficacious. Both assumptions might explain why antibiotic-resistant *N. gonorrhoeae* spread at somewhat higher rates in the dynamic transmission model than estimated from data. Third, we restricted our model to resistance to one antibiotic with no alternative treatment or interventions. This is why we observe complete replacement of the antibiotic-sensitive strain in the model, a phenomenon that has not been observed in surveillance data. Fourth, resistance in our model is treated as a generic trait, but it likely depends on the underlying molecular mechanisms and possibly the genetic background of the *N. gonorrhoeae* strain. Different resistance mechanisms might explain some of the differences in the rates of resistance spread between the model and the different antibiotics from the surveillance data. Fifth, we did not include co- and superinfection with antibiotic-sensitive and -resistant *N. gonorrhoeae* strains. Since genetic typing provides evidence for mixed infections [32], it is worth speculating how they would affect the rate of spread from the transmission model. If antibiotic-sensitive and -resistant strains co-existed in a host and acted independently, we would not expect significant effects on the rate of spread. In contrast, if there was competition between the two strains within a host, the rate of spread would increase if the antibiotic-resistant strain outcompetes the -sensitive strain, and decrease otherwise. Sixth, we do not consider importation of resistance from another population. For example, importation of resistance from other countries might play a particularly important role during the early phase of resistance spread, when stochastic events can lead to extinction of the antibiotic-resistant strain. We expect that a high rate of importation of antibiotic resistance shortens the time to reach 5% resistance drastically, but that once the resistant strain is established in the population, importation hardly affects the rate of resistance spread. Finally, we assumed that the transmission probabilities per partnership and the durations of infection in the model represent average values for *N. gonorrhoeae* infections at different infection sites (urethral, pharyngeal, anal, cervical).

The estimated posterior distributions of the parameters fit within the range of previously used values, and provide some insights into sexual mixing and the natural history of *N. gonorrhoeae*. The sexual mixing coefficient tends to be assortative for both HMW and MSM host populations in our model. Quantifying the degree of sexual mixing is difficult and largely depends on the study population, but our finding is consistent with other studies indicating assortative sexual mixing in the general population [30, 33]. The posterior estimates of the fraction of diagnosed and treated infections are consistent with the notion that a large proportion of *N. gonorrhoeae* infections are symptomatic, and that this proportion is expected to be higher in men than in women [34–36]. The average duration of infection was the only parameter with an informative prior, but we found marked differences between the duration of infection in HMW (6.6 months) and MSM (2.3 months). Per sex act transmission probabilities are generally considered to be lower from women to men than vice versa [37–39]. In our model, the median of the transmission probability per partnership was lower in MSM hosts than in HMW for both sexual activity groups. This could be explained by different numbers of sex acts per partnership in the two populations. The low transmission probability within the highly active MSM group (median: 30%) could reflect a single or a small number of sex acts per partnership. In contrast, the high transmission probability for HMW within the low sexual activity group (median: 87%) could be a result of a larger number of sex acts per partnership in those individuals. Furthermore, condom use is more frequent in MSM than in HMW [22], which could explain part of the observed differences in transmission probabilities.

Our study found that the treatment rate is the driving force of resistance spread. Xiridou et al. [13] found that resistance could spread faster when the treatment rate was higher, but they did not identify the treatment as the major driver of resistance spread. Chan et al. [12] found that focusing treatment on the core group leads to a faster rebound to pre-treatment prevalence than equal treatment of the entire host population. Unfortunately, our findings cannot be compared with Chan et al. because they do not report the proportion of antibiotic-resistant *N. gonorrhoeae*.

It was shown previously that treatment is the main selective force acting on resistance evolution due to the selective advantage to the resistant pathogen [40, 41]. We now expand this concept by showing that, assuming no fitness costs, treatment rates determine the rates of resistance spread even when the host populations has a heterogeneous contact structure. The intuitive argument that a faster spread of an infection, due to a higher number of sexual partners, will result in a faster spread of resistance does not hold. Instead, the proportion of resistant infections spreads equally in host populations with different number of partners as long as they receive treatment at the same rate and there are no fitness costs associated with the transmission probability per partnership. For *N. gonorrhoeae*, this insight challenges the current management strategy that aims to lower the overall burden of infection by expanding screening and treatment of hosts [9, 10]. As soon as antibiotic-resistant pathogens are frequent enough to evade stochastic extinction, expanded treatment will foster their spread and increase the burden of *N. gonorrhoeae*. Additionally, we show that fitness costs can decelerate or even prevent the spread of antibiotic-resistant *N. gonorrhoeae* strains. Fitness costs therefore might explain why highly resistant strains, such as the ceftriaxone-resistant *N. gonorrhoeae* strain H041, do not spread in the host population after their first detection [42]. Our findings also show that bridging between the HetM and the MSM host populations might not have been necessary for cefixime-resistance to spread in the HetM population after 2010 [5]. It is likely that cefixime-resistant *N. gonorrhoeae* had already been present in the HetM population but were spreading at a lower rate than in the MSM population.

The results of our study will be useful for future *N. gonorrhoeae* research and for guiding treatment recommendations. The *N. gonorrhoeae* transmission model describes observed prevalence and incidence rates well and can reconstruct the spread of antibiotic-resistant *N. gonorrhoeae*. Estimating rates of resistance spread is useful for projecting future resistance levels and the expected time it will take until a certain threshold in the proportion of antibiotic-resistant *N. gonorrhoeae* is reached. Until now, treatment recommendations for *N. gonorrhoeae* are subject to change when 5% of *N. gonorrhoeae* isolates show resistance against a given antibiotic [6]. Our study shows the importance of the rate of spread: a level of 5% resistance results in a marginal increase to 8% in the following year if resistance spreads logistically at rate 0.53 y^−1^ (HetM mean estimate from Table 4), but reaches 18% in the next year if resistance spreads at rate 1.46 y^−1^ (MSM mean estimate from Table 4). Public health authorities could use surveillance data and adapt thresholds for treatment recommendation change to specific host populations using the method we describe. Our study challenges the currently prevailing notion that more screening and treatment will limit the spread of *N. gonorrhoeae*, as higher treatment rates will ultimately result in faster spread of antibiotic resistance. Future treatment recommendations for *N. gonorrhoeae* should carefully balance prevention of *N. gonorrhoeae* infection and avoidance of the spread of resistance.

## Supporting Information

S1 Table Digitized data from the Gonococcal Resistance to Antimicrobials Surveillance Programme (GRASP). Data for heterosexual men (HetM) and men who have sex with men (MSM).
(PDF)

S2 Table Digitized data from the Gonococcal Isolate Surveillance Project (GISP). Data for men who have sex with women (MSW) and men who have sex with men (MSM). We used the MSW data as heterosexual men (HetM) data.
(PDF)

S3 Table Prevalence and incidence of diagnosed and treated infections after model calibration. (PDF)

S1 Fig Posterior distributions of (a) prevalence and (b) incidence of diagnosed and treated infections for MSM. (TIFF)

S2 Fig Posterior distributions of (a) prevalence and (b) incidence of diagnosed and treated infections for HMW. (TIFF)

S3 Fig Sensitivity of time to 5% resistance towards changes in the probability of resistance during treatment, *μ*. The time to 5% resistance of both MSM (green) and HMW (blue) are sensitive towards *μ*. Lower and upper bound of the box indicate the first and third quartiles, bar in the box indicates median, whiskers span 1.5 times IQR. Outliers are shown in orange and are outside 1.5 times IQR.
(TIFF)

S4 Fig Sensitivity of rate of spread towards changes in the probability of resistance during treatment, *μ*. The rates of spread of both MSM (green) and HMW (blue) are only sensitive towards *μ* when *μ* is unrealistically high. Lower and upper bound of the box indicate the first and third quartiles, bar in the box indicates median, whiskers span 1.5 times IQR. Outliers are shown in orange and are outside 1.5 times IQR.
(TIFF)

S1 Appendix Fitness costs and spread of resistance. (PDF)

## References

[1] Unemo M, Shafer WM. Antimicrobial Resistance in Neisseria gonorrhoeae in the 21st Century: Past, Evolution, and Future. Clin Microbiol Rev. 2014 7;27(3):587–613. Available from: http://www.ncbi.nlm.nih.gov/pubmed/24982323. 10.1128/CMR.00010-14 24982323 PMC4135894

[2] Centers for Disease Control and Prevention. Sexually Transmitted Disease Surveillance 2012. Atlanta: U.S. Department of Health and Human Services; 2014.

[3] Public Health England. GRASP 2013 Report The Gonococcal Resistance to Antimicrobials Surveillance Programme (England and Wales); 2014.

[4] Davies S, Fowler T, Watson J, Livermore D, Walker D. Annual Report of the Chief Medical Officer: infection and the rise of antimicrobial resistance. Lancet. 2013;381:1606–1609. Available from: http://www.thelancet.com/journals/lancet/article/PIIS0140-6736(13)60604-2/abstract. 10.1016/S0140-6736(13)60604-2 23489756

[5] Ison CA, Town K, Obi C, Chisholm S, Hughes G, Livermore D, et al Decreased susceptibility to cephalosporins among gonococci: data from the Gonococcal Resistance to Antimicrobials Surveillance Programme (GRASP) in England and Wales, 2007–2011. Lancet Infect Dis. 2013 9;13(9):762–8. Available from: http://www.ncbi.nlm.nih.gov/pubmed/23764300. 10.1016/S1473-3099(13)70143-9 23764300

[6] Tapsall J, World Health Organization. Antimicrobial resistance in Neisseria gonorrhoeae; 2001. Available from: http://www.who.int/entity/csr/resources/publications/drugresist/Neisseria_gonorrhoeae.pdf.

[7] Bignell C, Unemo M. 2012 European Guideline on the Diagnosis and Treatment of Gonorrhoea in Adults. Int J STD AIDS. 2013 2;24(2):85–92. Available from: http://www.ncbi.nlm.nih.gov/pubmed/24400344. 10.1177/0956462412472837 24400344

[8] Kirkcaldy RD, Weinstock HS, Moore PC, Philip SS, Wiesenfeld HC, Papp JR, et al The Efficacy and Safety of Gentamicin Plus Azithromycin and Gemifloxacin Plus Azithromycin as Treatment of Uncomplicated Gonorrhea. Clin Infect Dis. 2014 10;59(8):1083–91. Available from: http://www.ncbi.nlm.nih.gov/pubmed/25031289. 10.1093/cid/ciu521 25031289 PMC4271098

[9] Campos-Outcalt D. CDC update on gonorrhea: Expand treatment to limit resistance. J Fam Practice. 2011;60(12):736–739. 22163356

[10] Centers for Disease Control and Prevention. Cephalosporin-resistant Neisseria gonorrhoeae Public Health Response Plan; 2012 Available from: http://www.cdc.gov/std/treatment/ceph-r-responseplanjuly30-2012.pdf.

[11] Yorke J, Hethcote H, Nold A. Dynamics and Control of the Transmission of Gonorrhea. Sex Transm Dis. 1978;5(2):51–56. Available from: http://journals.lww.com/stdjournal/Abstract/1978/04000/Dynamics_and_Control_of_the_Transmission_of.3.aspx. 10328031 10.1097/00007435-197804000-00003

[12] Chan CH, McCabe CJ, Fisman DN. Core groups, antimicrobial resistance and rebound in gonorrhoea in North America. Sex Transm Infect. 2012 4;88(3):200–4. Available from: http://www.ncbi.nlm.nih.gov/pubmed/22169277. 10.1136/sextrans-2011-050049 22169277

[13] Xiridou M, Soetens LC, Koedijk FDH, van der Sande MAB, Wallinga J. Public health measures to control the spread of antimicrobial resistance in Neisseria gonorrhoeae in men who have sex with men. Epidemiol Infect. 2015 10;143(8):1575–1584. Available from: http://www.ncbi.nlm.nih.gov/pubmed/25275435. 10.1017/S0950268814002519 25275435 PMC9507228

[14] Hui BB, Ryder N, Su JY, Ward J, Chen MY, Donovan B, et al Exploring the Benefits of Molecular Testing for Gonorrhoea Antibiotic Resistance Surveillance in Remote Settings. PloS One. 2015;10(7):e0133202 10.1371/journal.pone.0133202 26181042 PMC4504484

[15] Health Protection Agency. GRASP 2011 Report: The Gonococcal Resistance to Antimicrobials Surveillance Programme. 2012;p. 12 Available from: http://webarchive.nationalarchives.gov.uk/20140714084352/http://www.hpa.org.uk/webc/HPAwebFile/HPAweb_C/1317136030908.

[16] Health Protection Agency. GRASP 2011 Report: The Gonococcal Resistance to Antimicrobials Surveillance Programme. 2012;p. 14 Available from: http://webarchive.nationalarchives.gov.uk/20140714084352/http://www.hpa.org.uk/webc/HPAwebFile/HPAweb_C/1317136030908.

[17] Centers for Disease Control and Prevention. Sexually Transmitted Disease Surveillance 2011. 2012;p. 54 Available from: http://www.cdc.gov/std/stats11/surv2011.pdf.

[18] Plot Digitizer 2.6.6. 2014;Available from: http://plotdigitizer.sourceforge.net/.

[19] R Core Team. R: A Language and Environment for Statistical Computing. Vienna, Austria; 2015 Available from: http://www.r-project.org/.

[20] Romero-Severson EO, Alam SJ, Volz EM, Koopman JS. Heterogeneity in Number and Type of Sexual Contacts in a Gay Urban Cohort. Stat Commun Infect Dis. 2012;4(1). Available from: http://www.pubmedcentral.nih.gov/articlerender.fcgi?artid=3639013&tool=pmcentrez&rendertype=abstract. 10.1515/1948-4690.1042 PMC3639013 23638243

[21] Garnett GP, Swinton J, Brunham RC, Anderson RM. Gonococcal infection, infertility, and population growth: II. The influence of heterogeneity in sexual behaviour. IMA J Math Appl Med Biol. 1992;9(2):127–144. 10.1093/imammb/9.2.127 1517674

[22] Johnson AM, Mercer CH, Erens B, Copas AJ, McManus S, Wellings K, et al Sexual behaviour in Britain: Partnerships, practices, and HIV risk behaviours. Lancet. 2001;358(9296):1835–1842. 10.1016/S0140-6736(01)06883-0 11741621

[23] Althaus CL, Heijne JCM, Herzog SA, Roellin A, Low N. Individual and Population Level Effects of Partner Notification for Chlamydia trachomatis. PloS One. 2012 1;7(12):e51438 Available from: http://www.pubmedcentral.nih.gov/articlerender.fcgi?artid=3520891&tool=pmcentrez&rendertype=abstract. 10.1371/journal.pone.0051438 23251534 PMC3520891

[24] Soetaert, K. rootSolve: Nonlinear root finding, equilibrium and steady-state analysis of ordinary differential equations; 2009. Available from: https://cran.r-project.org/web/packages/rootSolve/.

[25] Garnett GP, Mertz KJ, Finelli L, Levine WC, Louis ME St. The transmission dynamics of gonorrhoea: modelling the reported behaviour of infected patients from Newark, New Jersey. Philos Trans R Soc Lond B Biol Sci. 1999 4;354(1384):787–97. Available from: http://www.pubmedcentral.nih.gov/articlerender.fcgi?artid=1692556&tool=pmcentrez&rendertype=abstract. 10.1098/rstb.1999.0431 10365404 PMC1692556

[26] Datta SD, Sternberg M, Johnson RE, Berman S, Papp JR, McQuillan G, et al Gonorrhea and Chlamydia in the United States among Persons 14 to 39 Years of Age, 1999 to 2002. Ann Intern Med. 2007;147(2):89–96. 10.7326/0003-4819-147-2-200707170-00007 17638719

[27] Centers for Disease Control and Prevention. Sexually Transmitted Disease Surveillance 2013. Atlanta: U.S. Department of Health and Human Services; 2014.

[28] Jin F, Prestage GP, Mao L, Kippax SC, Pell CM, Donovan B, et al Incidence and risk factors for urethral and anal gonorrhoea and chlamydia in a cohort of HIV-negative homosexual men: the Health in Men Study. Sex Transm Infect. 2007;83(2):113–119. 10.1136/sti.2006.021915 17005541 PMC2598603

[29] Jin F. Personal Communication. 2015 may.

[30] Althaus CL, Choisy M, Alizon S, CSF group. Number of sex acts matters for heterosexual transmission and control of Chlamydia trachomatis. PeerJ PrePrints. 2015;3:e1164 Available from: 10.7287/peerj.preprints.940v1.

[31] Grad YH, Goldstein E, Lipsitch M, White PJ. Improving Control of Antibiotic-Resistant Gonorrhea by Integrating Research Agendas Across Disciplines: Key Questions Arising From Mathematical Modeling. J Infect Dis. 2016;213(6):883–890. Available from: http://jid.oxfordjournals.org/content/213/6/883. 10.1093/infdis/jiv517 26518045 PMC4760416

[32] Lynn F, Hobbs MM, Zenilman JM, Behets FMTF, Van Damme K, Rasamindrakotroka A, et al Genetic typing of the porin protein of Neisseria gonorrhoeae from clinical noncultured samples for strain characterization and identification of mixed gonococcal infections. J Clin Microbiol. 2005;43(1):368–375. 10.1128/JCM.43.1.368-375.2005 15634996 PMC540152

[33] Renton A, Whitaker L, Ison C, Wadsworth J, Harris JRW. Estimating the sexual mixing patterns in the general population from those in people acquiring gonorrhoea infection: theoretical foundation and empirical findings. J Epidemiol Community Health. 1995 4;49(2):205–213. Available from: http://jech.bmj.com/cgi/doi/10.1136/jech.49.2.205. 10.1136/jech.49.2.205 7798052 PMC1060109

[34] Farley TA, Cohen DA, Elkins W. Asymptomatic sexually transmitted diseases: the case for screening. Prev Med. 2003;36(4):502–509. Available from: http://www.sciencedirect.com/science/article/pii/S0091743502000580. 10.1016/S0091-7435(02)00058-0 12649059

[35] Buimer M, van Doornum GJ, Ching S, Peerbooms PG, Plier PK, Ram D, et al Detection of Chlamydia trachomatis and Neisseria gonorrhoeae by ligase chain reaction-based assays with clinical specimens from various sites: implications for diagnostic testing and screening. J Clin Microbiol. 1996;34(10):2395–400. Available from: http://www.pubmedcentral.nih.gov/articlerender.fcgi?artid=229278&tool=pmcentrez&rendertype=abstract. 8880487 10.1128/jcm.34.10.2395-2400.1996 PMC229278

[36] Chen MI, Ghani AC, Edmunds WJ. A metapopulation modelling framework for gonorrhoea and other sexually transmitted infections in heterosexual populations. J R Soc Interface. 2009 9;6(38):775–791. Available from: http://rsif.royalsocietypublishing.org/cgi/doi/10.1098/rsif.2008.0394. 10.1098/rsif.2008.0394 18986961 PMC2855508

[37] Hethcote H, Yorke J. Gonorrhea Transmission Dynamics and Control. Lecture Notes in Bioinformatics. 1984;56 10.1007/978-3-662-07544-9

[38] Swinton J, Garnett GP, Brunham RC, Anderson RM. Gonococcal infection, infertility, and population growth: I. Endemic states in behaviourally homogeneous growing populations. IMA J Math Appl Med Biol. 1992;9(2):107–126. 10.1093/imammb/9.2.107 1517673

[39] Bracher M, Watkins S, Santow G. “Moving” and Marrying: Modelling HIV Infection among Newly-weds in Malawi. Demogr Res. 2003 9;Special 1:207–246. Available from: http://www.demographic-research.org/special/1/7/. 10.4054/DemRes.2003.S1.7

[40] Bonhoeffer S, Lipsitch M, Levin BR. Evaluating treatment protocols to prevent antibiotic resistance. Proc Natl Acad Sci USA. 1997 10;94(22):12106–11. Available from: http://www.ncbi.nlm.nih.gov/pubmed/9342370. 10.1073/pnas.94.22.12106 9342370 PMC23718

[41] Huijben S, Bell AS, Sim DG, Tomasello D, Mideo N, Day T, et al Aggressive Chemotherapy and the Selection of Drug Resistant Pathogens. PLoS Pathog. 2013 9;9(9):e1003578 10.1371/journal.ppat.1003578 24068922 PMC3771897

[42] Shimuta K, Unemo M, Nakayama SI, Morita-Ishihara T, Dorin M, Kawahata T, et al Antimicrobial Resistance and Molecular Typing of Neisseria gonorrhoeae Isolates in Kyoto and Osaka, Japan, 2010 to 2012: Intensified Surveillance after Identification of the First Strain (H041) with High-Level Ceftriaxone Resistance. Antimicrob Agents Chemother. 2013 11;57(11):5225–32. Available from: http://www.ncbi.nlm.nih.gov/pubmed/23939890 http://www.pubmedcentral.nih.gov/articlerender.fcgi?artid=PMC3811299. 10.1128/AAC.01295-13 23939890 PMC3811299

