# Supplementary material for: Antibiotic-Resistant Neisseria gonorrhoeae Spread Faster with More Treatment, Not More Sexual Partners
Source: PLoS Pathog. 2016 May 19;12(5):e1005611. doi: 10.1371/journal.ppat.1005611 (PMC4872991; doi:10.1371/journal.ppat.1005611)
Supplement: S1 Table — Data for heterosexual men (HetM) and men who have sex with men (MSM). (PDF) [file ppat.1005611.s001.pdf]

S1 Table. Digitized data from the Gonococcal Resistance to Antimicrobials Surveillance Programme (GRASP). Data for heterosexual men (HetM) and men who have sex with men (MSM).

Year	Resistance	Programme	Population	Drug
2004	0.00	GRASP	HetM	Cefixime
2005	0.00	GRASP	HetM	Cefixime
2006	0.00	GRASP	HetM	Cefixime
2007	0.41	GRASP	HetM	Cefixime
2008	1.42	GRASP	HetM	Cefixime
2009	2.23	GRASP	HetM	Cefixime
2010	5.69	GRASP	HetM	Cefixime
2004	0.00	GRASP	MSM	Cefixime
2005	0.00	GRASP	MSM	Cefixime
2006	0.20	GRASP	MSM	Cefixime
2007	2.94	GRASP	MSM	Cefixime
2008	5.18	GRASP	MSM	Cefixime
2009	24.06	GRASP	MSM	Cefixime
2010	33.40	GRASP	MSM	Cefixime
2000	3.06	GRASP	HetM	Ciprofloxacin
2001	4.59	GRASP	HetM	Ciprofloxacin
2002	12.13	GRASP	HetM	Ciprofloxacin
2003	10.80	GRASP	HetM	Ciprofloxacin
2004	10.21	GRASP	HetM	Ciprofloxacin
2005	11.10	GRASP	HetM	Ciprofloxacin
2006	18.49	GRASP	HetM	Ciprofloxacin
2007	21.11	GRASP	HetM	Ciprofloxacin
2008	24.66	GRASP	HetM	Ciprofloxacin
2009	28.49	GRASP	HetM	Ciprofloxacin
2000	0.76	GRASP	MSM	Ciprofloxacin
2001	2.01	GRASP	MSM	Ciprofloxacin
2002	8.50	GRASP	MSM	Ciprofloxacin
2003	10.61	GRASP	MSM	Ciprofloxacin
2004	26.27	GRASP	MSM	Ciprofloxacin
2005	42.52	GRASP	MSM	Ciprofloxacin
2006	43.13	GRASP	MSM	Ciprofloxacin
2007	47.41	GRASP	MSM	Ciprofloxacin
2008	45.83	GRASP	MSM	Ciprofloxacin
2009	53.88	GRASP	MSM	Ciprofloxacin
