# Supplementary material for: Antibiotic-Resistant Neisseria gonorrhoeae Spread Faster with More Treatment, Not More Sexual Partners
Source: PLoS Pathog. 2016 May 19;12(5):e1005611. doi: 10.1371/journal.ppat.1005611 (PMC4872991; doi:10.1371/journal.ppat.1005611)
Supplement: S2 Table — Data for men who have sex with women (MSW) and men who have sex with men (MSM). We used the MSW data as heterosexual men (HetM) data. (PDF) [file ppat.1005611.s002.pdf]

**S2 Table. Digitized data from the Gonococcal Isolate Surveillance Project (GISP).** Data for men who have sex with women (MSW) and men who have sex with men (MSM). We used the MSW data as heterosexual men (HetM) data.

| Year | Resistance | Programme | Population | Drug |
|---|---|---|---|---|
| 1995 | 0.35 | GISP | MSW | Ciprofloxacin |
| 1996 | 0.18 | GISP | MSW | Ciprofloxacin |
| 1997 | 0.27 | GISP | MSW | Ciprofloxacin |
| 1998 | 0.24 | GISP | MSW | Ciprofloxacin |
| 1999 | 0.50 | GISP | MSW | Ciprofloxacin |
| 2000 | 0.41 | GISP | MSW | Ciprofloxacin |
| 2001 | 0.56 | GISP | MSW | Ciprofloxacin |
| 2002 | 1.00 | GISP | MSW | Ciprofloxacin |
| 2003 | 1.59 | GISP | MSW | Ciprofloxacin |
| 2004 | 2.86 | GISP | MSW | Ciprofloxacin |
| 2005 | 3.86 | GISP | MSW | Ciprofloxacin |
| 2006 | 7.04 | GISP | MSW | Ciprofloxacin |
| 1995 | 0.09 | GISP | MSM | Ciprofloxacin |
| 1996 | 0.06 | GISP | MSM | Ciprofloxacin |
| 1997 | 0.41 | GISP | MSM | Ciprofloxacin |
| 1998 | 0.03 | GISP | MSM | Ciprofloxacin |
| 1999 | 0.47 | GISP | MSM | Ciprofloxacin |
| 2000 | 0.62 | GISP | MSM | Ciprofloxacin |
| 2001 | 1.65 | GISP | MSM | Ciprofloxacin |
| 2002 | 7.31 | GISP | MSM | Ciprofloxacin |
| 2003 | 15.09 | GISP | MSM | Ciprofloxacin |
| 2004 | 23.92 | GISP | MSM | Ciprofloxacin |
| 2005 | 29.11 | GISP | MSM | Ciprofloxacin |
| 2006 | 39.13 | GISP | MSM | Ciprofloxacin |
