# Supplementary material for: Antibiotic-Resistant Neisseria gonorrhoeae Spread Faster with More Treatment, Not More Sexual Partners
Source: PLoS Pathog. 2016 May 19;12(5):e1005611. doi: 10.1371/journal.ppat.1005611 (PMC4872991; doi:10.1371/journal.ppat.1005611)
Supplement: S3 Table — (PDF) [file ppat.1005611.s003.pdf]

**S3 Table. Prevalence and incidence of diagnosed and treated infections after model calibration.**

| Measure | Population | sexual activity | Median | IQR |
|---|---|---|---|---|
| Prevalence (in %) | HMW | low | 0.12 | 0.09-0.15 |
| Prevalence (in %) | HMW | high | 2.13 | 1.74-2.64 |
| Prevalence (in %) | HMW | either | 0.25 | 0.20-0.31 |
| Incidence (in % person$^{-1}$ y$^{-1}$) | HMW | low | 0.04 | 0.02-0.06 |
| Incidence (in % person$^{-1}$ y$^{-1}$) | HMW | high | 2.85 | 2.16-3.74 |
| Incidence (in % person$^{-1}$ y$^{-1}$) | HMW | either | 0.23 | 0.17-0.29 |
| Prevalence (in %) | MSM | low | 0.58 | 0.41-0.79 |
| Prevalence (in %) | MSM | high | 27.41 | 22.88-32.08 |
| Prevalence (in %) | MSM | either | 2.07 | 1.67-2.44 |
| Incidence (in % person$^{-1}$ y$^{-1}$) | MSM | low | 1.02 | 0.54-1.5 |
| Incidence (in % person$^{-1}$ y$^{-1}$) | MSM | high | 104.52 | 95.65-113.4 |
| Incidence (in % person$^{-1}$ y$^{-1}$) | MSM | either | 6.49 | 6.18-6.83 |
