# Supplementary figures and images for: Antibiotic-Resistant Neisseria gonorrhoeae Spread Faster with More Treatment, Not More Sexual Partners
Source: PLoS Pathog. 2016 May 19;12(5):e1005611. doi: 10.1371/journal.ppat.1005611 (PMC4872991; doi:10.1371/journal.ppat.1005611)

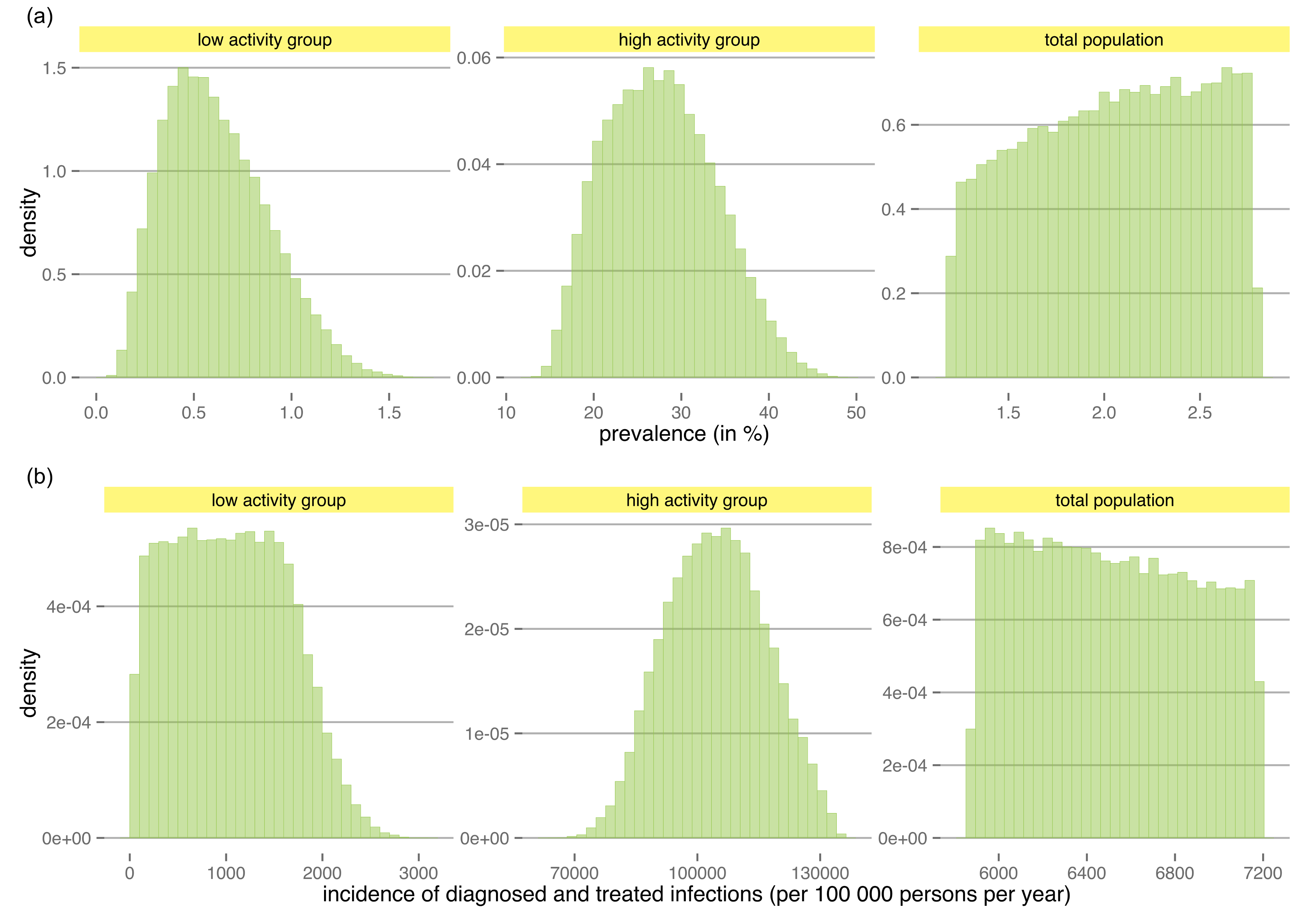

Supplement: S1 Fig — (TIFF) [file ppat.1005611.s004.tiff]

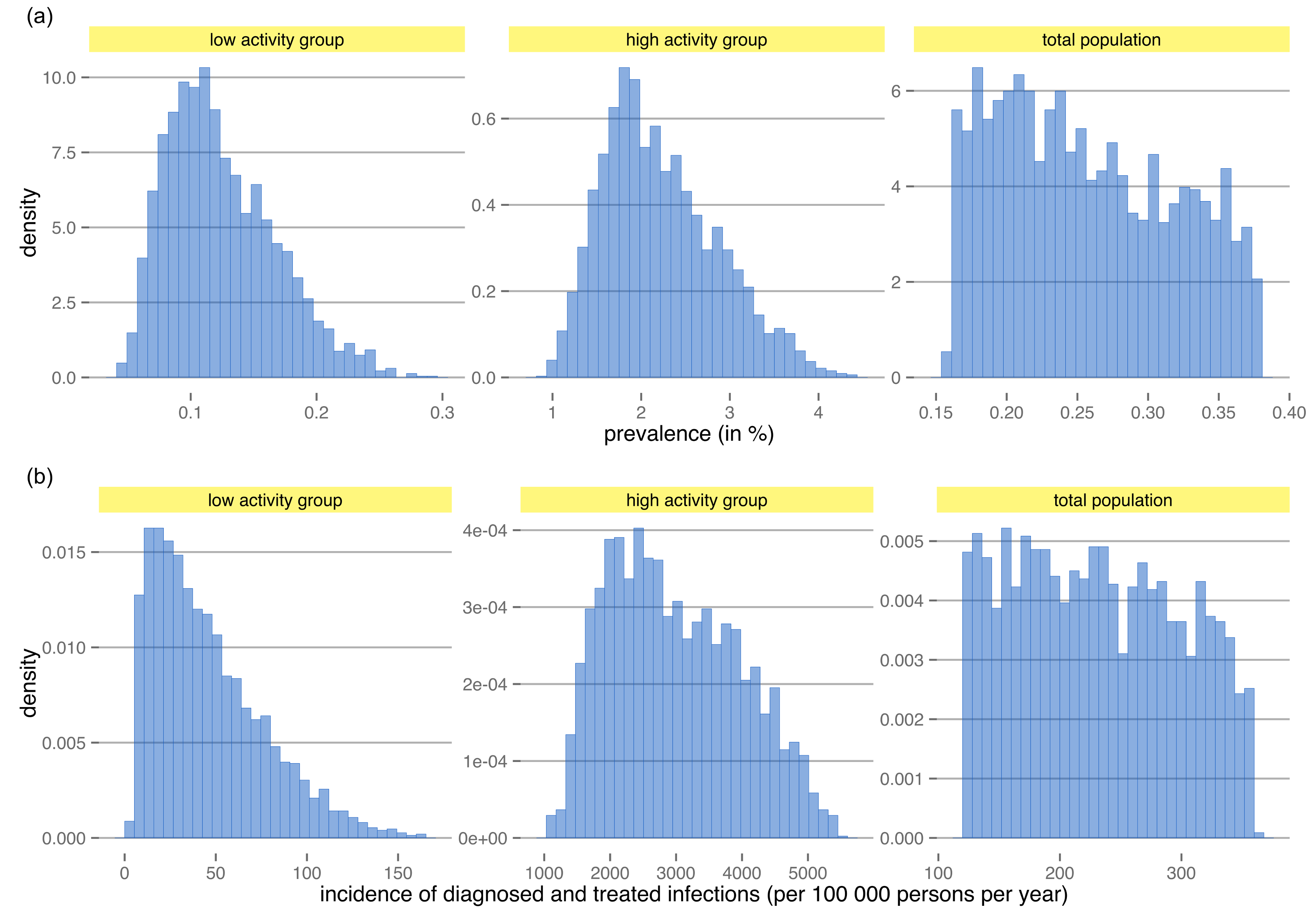

Supplement: S2 Fig — (TIFF) [file ppat.1005611.s005.tiff]

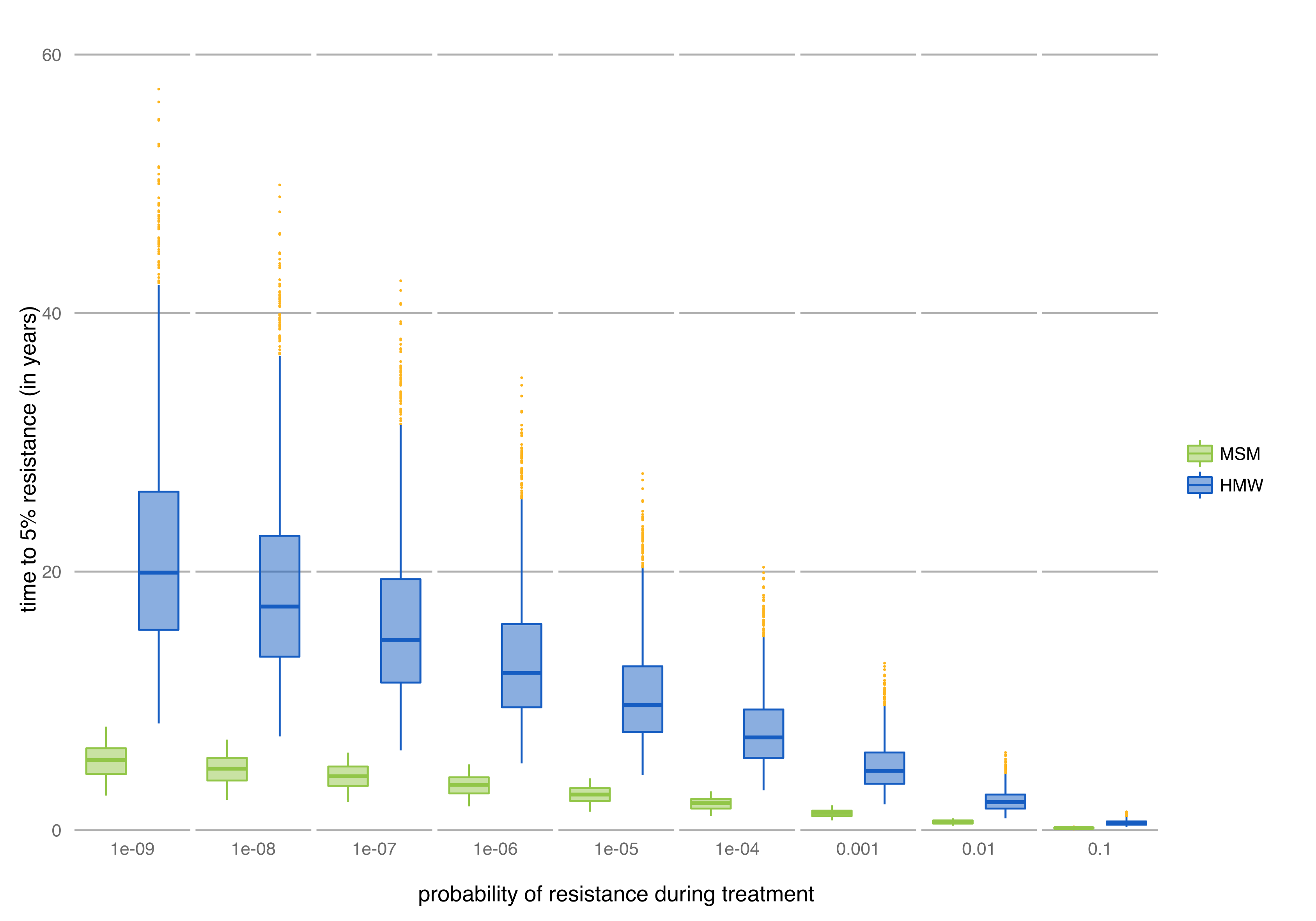

Supplement: S3 Fig — The time to 5% resistance of both MSM (green) and HMW (blue) are sensitive towards μ. Lower and upper bound of the box indicate the first and third quartiles, bar in the box indicates median, whiskers span 1.5 times IQR. Outliers are shown in orange and are outside 1.5 times IQR. (TIFF) [file ppat.1005611.s006.tiff]

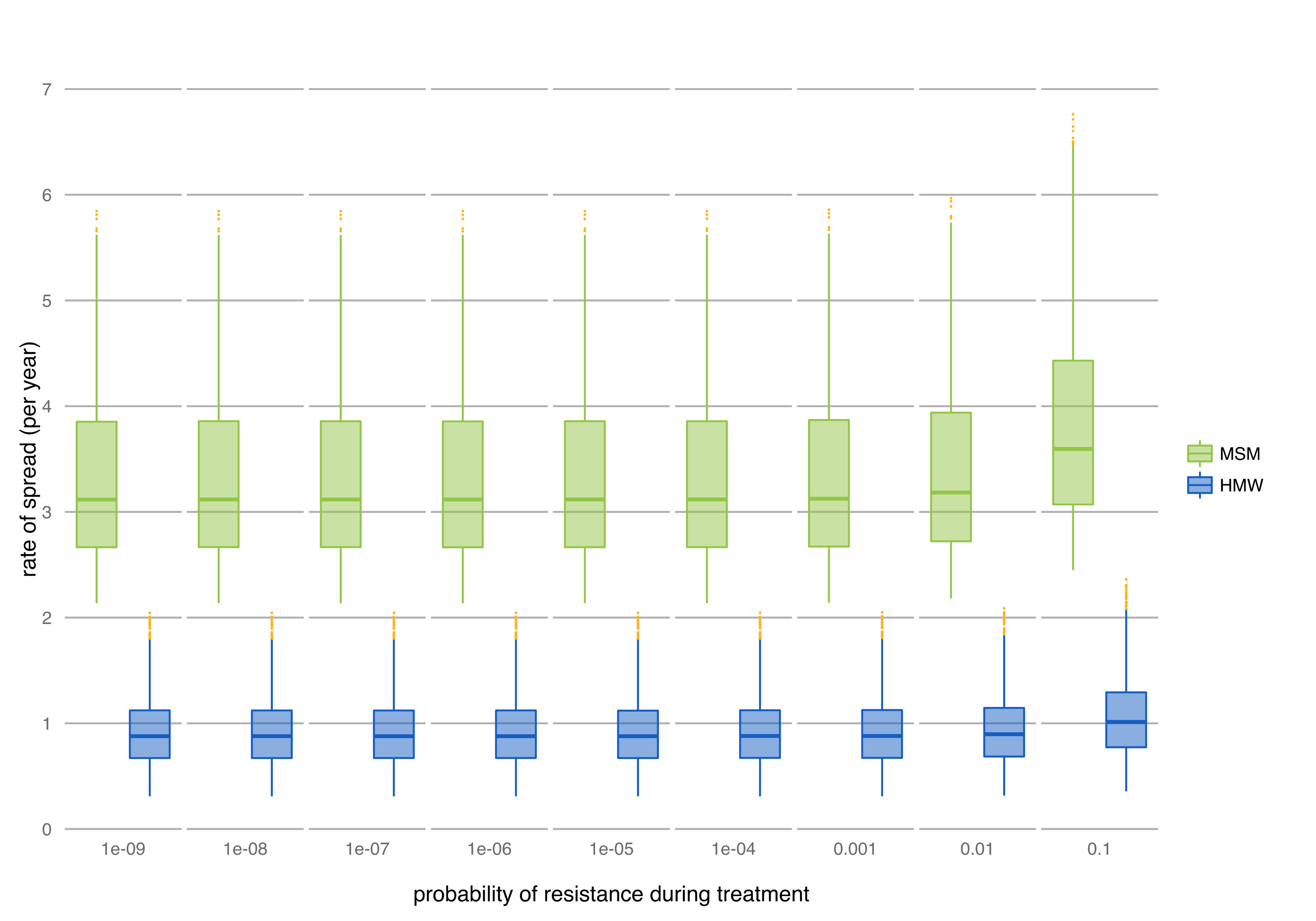

Supplement: S4 Fig — The rates of spread of both MSM (green) and HMW (blue) are only sensitive towards μ when μ is unrealistically high. Lower and upper bound of the box indicate the first and third quartiles, bar in the box indicates median, whiskers span 1.5 times IQR. Outliers are shown in orange and are outside 1.5 times IQR. (TIFF) [file ppat.1005611.s007.tiff]
