# Supplementary material for: Antibiotic-Resistant Neisseria gonorrhoeae Spread Faster with More Treatment, Not More Sexual Partners
Source: PLoS Pathog. 2016 May 19;12(5):e1005611. doi: 10.1371/journal.ppat.1005611 (PMC4872991; doi:10.1371/journal.ppat.1005611)
Supplement: S1 Appendix — (PDF) [file ppat.1005611.s008.pdf]

Antibiotic-resistant *Neisseria gonorrhoeae* spread faster with more treatment

Supporting Information - S1 Appendix

Stephanie M. Fingerhuth[1,2,*], Sebastian Bonhoeffer[1], Nicola Low[2], Christian L. Althaus[2]

1 Institute of Integrative Biology, ETH Zurich, Zurich, Switzerland
2 Institute of Social and Preventive Medicine (ISPM), University of Bern, Bern, Switzerland

*** stephanie.fingerhuth@env.ethz.ch**

Fitness costs and spread of resistance

We investigated how fitness costs affect the spread of antibiotic-resistant *Neisseria gonorrhoeae*. In the obligate human pathogen *N. gonorrhoeae*, fitness costs can affect the transmission probability of the pathogen and the duration of *N. gonorrhoeae* infection. Therefore we included fitness costs in both transmission probability per partnership and duration of infection in our model (for details see Model Extension and Simulation section below).

We simulated our model and found that the proportion of simulations with successful resistance spread decreases with increasing fitness costs (Fig. A). The rates of spread also decrease with increasing fitness costs (Fig. B). We analytically approximated the rate of spread of antibiotic-resistant *N. gonorrhoeae* that suffer from fitness costs in the duration of infection and found the obtained approximation to be in agreement with the simulation results. We evaluated whether fitness costs affect low and high activity groups differently. The relative difference in rate of spread between activity groups fluctuates around zero when fitness costs in the duration of infection are simulated (Fig. Ca, Cc), but it changes with fitness costs in the transmission probability per partnership (Fig. Cb, Cd). This means that resistance spreads at the same rate in both activity groups when the duration of infection is affected by fitness costs. When the fitness costs affect the transmission probability per partnership, resistance spreads differently between activity groups, because they differ in the sexual partner change rate, π_i, and the transmission probability within the activity group, β_{ii}. It is noteworthy that the proportion of unsuccessful spread and the relative difference of rate of spread between activity groups is small for small fitness costs.

Model Extension and Simulation

First, we assumed fitness cost ω_β leads to a relative reduction in the transmission probability per partnership:

$$\beta_{ij_{Res}} = \beta_{ij}(1 - \omega_\beta),$$

where β_{ij} and $\beta_{ij_{Res}}$ are the transmission probabilities per partnership of the antibiotic-sensitive and -resistant *N. gonorrhoeae* strains. Second, we assumed fitness cost ω_ν leads to a relative reduction in the average duration of infection. Since we

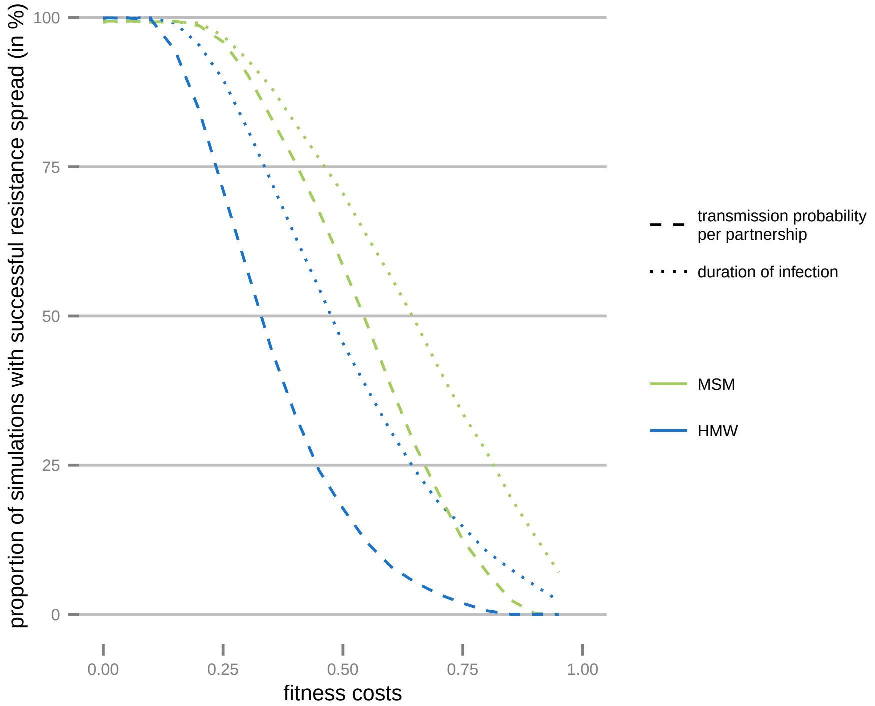

Figure A. Fitness costs in the antibiotic-resistant strain can prevent the spread of resistance. Fitness costs have a larger effect in preventing the spread of resistance in HMW (blue) than in MSM (green). Similarly, the effect is stronger when they act on the transmission probability per partnership than the duration of infection.

assume resistance is complete, the duration of infection of the antibiotic-resistant strain is independent of the treatment rate τ and thus

$$D_{Res} = \frac{1}{\nu_{Res}} = \frac{1}{\nu}(1 - \omega_\nu),$$

which gives

$$\nu_{Res} = \frac{\nu}{1 - \omega_\nu},$$

where D_{Res} is the average duration of infection of the antibiotic-resistant strain, and ν and ν_{Res} are the spontaneous recovery rates from the antibiotic-sensitive and -resistant strain.

The rate at which the antibiotic-resistant strain replaces the -sensitive strain is given by the difference in their net growth rates, $\Delta\psi$. Assuming that the antibiotic-resistant strain only carries a fitness cost that affects the duration of infection, the rate of resistance spread is approximated by

$$\Delta\psi = \tau + \nu - \frac{\nu}{1 - \omega_\nu} = \tau - \nu\frac{\omega_\nu}{1 - \omega_\nu}.$$

For ω_ν = 0, the rate of resistance spread is then approximated by $\Delta\psi = \tau$ as described in the main text. Deriving an analytical solution for the difference in the net growth rates when the fitness costs affect the transmission probability per partnership is less trivial, since the two strains then have different forces of infection.

We simulated the model with a subset of 2000 calibrated parameter sets each for men who have sex with men (MSM) and heterosexual men and women (HMW). We

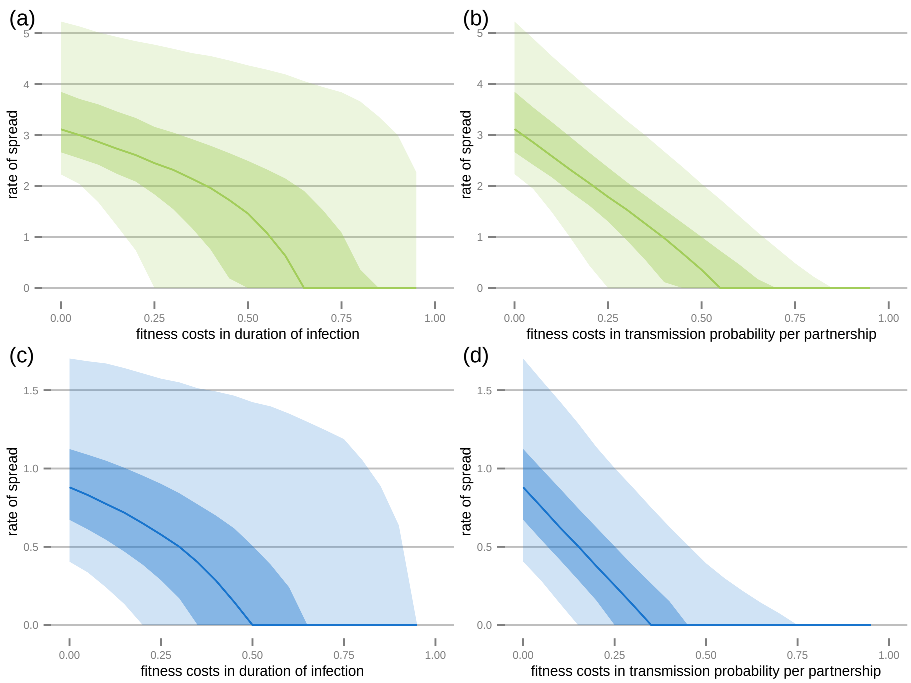

Figure B. Fitness costs in the antibiotic-resistant strain decrease the rate of resistance spread. (a,c) Fitness cost affects the duration of infection. (b,d) Fitness cost affects the transmission probability per partnership. HMW and MSM are shown in blue and green. The solid lines correspond to the median rates of resistance spread for all simulations. The 50% and 95% intervals are shown in dark and light color.

simulated fitness costs from 0 to 95% in either transmission probability per partnership or duration of infection. We fit the simulated proportion of resistant *N. gonorrhoeae* to logistic growth models using the least squares function *nls* and *SSlogis* in R. When fitting was unsuccessful or the estimated asymptote was smaller than 99%, i.e. the proportion resistant did not fixate in the population, we assumed that the spread of resistance was unsuccessful and set the rate of spread to zero. We calculated the relative differences in rate of spread between activity groups with

$$\frac{\text{rate of spread in the low activity group} - \text{rate of spread in the high activity group}}{\text{rate of spread in overall population}}$$

to evaluate whether fitness costs affect the activity groups differently.

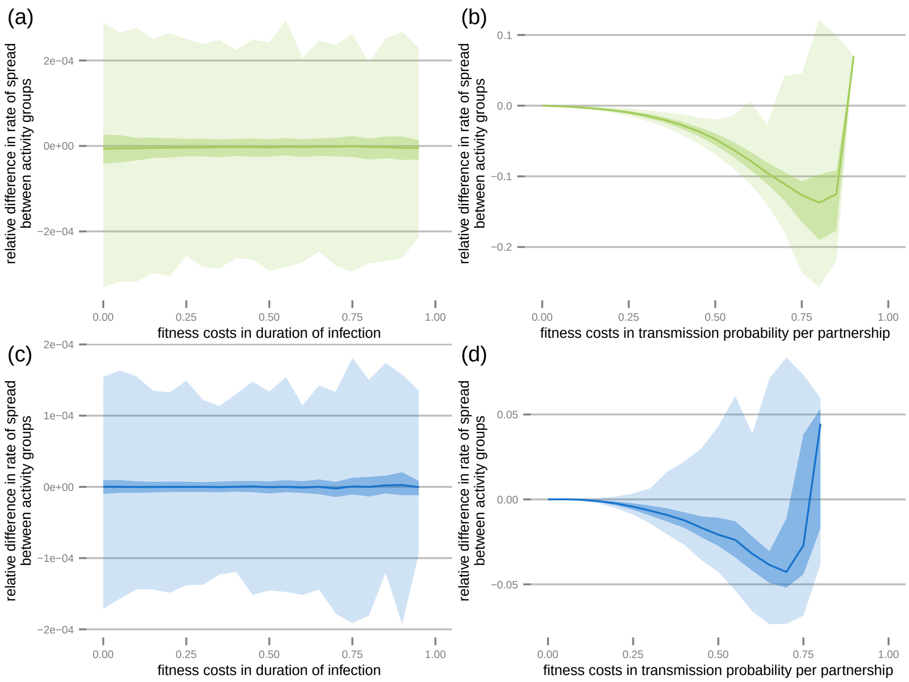

Figure C. Relative difference in rate of spread between activity groups and fitness costs. The relative difference in rate of spread fluctuates around 0 in MSM (green) and HMW (blue) when the fitness costs affect the duration of infection (a,c), but changes with fitness costs affecting the transmission probability per partnership (b,d). Shown are median (line), interval including 50% (dark color), interval including 95% (light color) of relative difference in rates of spread. See text for calculation of relative difference in rate of spread.
